# Geographic weighted regression analysis of hot spots of anemia and its associated factors among children aged 6–59 months in Ethiopia: A geographic weighted regression analysis and multilevel robust Poisson regression analysis

**DOI:** 10.1371/journal.pone.0259147

**Published:** 2021-11-04

**Authors:** Getayeneh Antehunegn Tesema, Zemenu Tadesse Tessema, Dessie Abebaw Angaw, Koku Sisay Tamirat, Achamyeleh Birhanu Teshale

**Affiliations:** Department of Epidemiology and Biostatistics, Institute of Public Health, College of Medicine and Health Sciences, University of Gondar, Gondar, Ethiopia; INRAE, FRANCE

## Abstract

**Introduction:**

Anemia among children aged 6–59 months remains a major public health problem in low-and high-income countries including Ethiopia. Anemia is associated with significant consequences on the health of children such as under-five morbidity and mortality, increased risk of infection, and poor academic performance. The prevalence of anemia in Ethiopia has varied across areas. Therefore, this study aimed to investigate the geographic weighted regression analysis of anemia and its associated factors among children aged 6–59 months in Ethiopia.

**Methods:**

This study was based on the 2016 Ethiopian Demographic and Health Survey (EDHS) data. A total weighted sample of 8482 children aged 6–59 months was included. For the spatial analysis, Arc-GIS version 10.7 and SaTScan version 9.6 statistical software were used. Spatial regression was done to identify factors associated with the hotspots of anemia and model comparison was based on adjusted R^2^ and Corrected Akaike Information Criteria (AICc). For the associated factors, the multilevel robust Poisson regression was fitted since the prevalence of anemia was greater than 10%. Variables with a p-value < 0.2 in the bi-variable analysis were considered for the multivariable analysis. In the multivariable multilevel robust Poisson regression analysis, the adjusted prevalence ratio with the 95% confidence interval was reported to declare the statistical significance and strength of association.

**Results:**

The prevalence of anemia among children aged 6–59 months was 57.56% (95%CI: 56.50%, 58.61%) with significant spatial variation across regions in Ethiopia. The significant hot spot areas of anemia among children aged 6–59 months were detected in the central, west, and east Afar, Somali, Dire Dawa, Harari, and northwest Gambella regions. Mothers who had anemia, a child aged 23–59 months, mothers aged 15–19 years, and coming from a household with a poorer or poorest household were significant predictors of the spatial variations of anemia among children aged 6–59 months. In the multilevel robust Poisson analysis, born to mothers aged 30–39 (APR = 0.84, 95% CI: 0.76, 0.92) and 40–49 years (APR = 0.73, 95% CI: 0.65, 0.83), mothers who didn’t have formal education (APR = 1.10, 95% CI: 1.00, 1.20), Children in the poorest household wealth index (APR = 1.17, 95% CI: 1.06, 1.29), being 4–6 (APR = 1.08, 95% CI: 1.02, 1.13) and above 6 order of birth (APR = 1.15, 95% CI: 1.07, 1.23), children born to anemic mothers (APR = 1.24, 95% CI: 1.19, 1.29), children aged 24–59 months (APR = 0.70, 95% CI: 0.68, 0.73), stunted children (APR = 1.09, 95% CI: 1.04, 1.13) and underweight children (APR = 1.07, 95% CI: 1.03, 1.13) were significantly associated with anemia among children aged 6–59 months.

**Conclusion and recommendation:**

Anemia is still a public health problem for children in Ethiopia. Residing in a geographic area where a high proportion of children born to mothers aged 15–19 years, a child aged 6–23 months, coming from a household with poorer or poorest wealth index, and mothers with anemia increased the risk of experiencing anemia among children aged 6–59 months. Maternal education, maternal age, child age, household wealth, stunting, underweight, birth order, and maternal anemia were significant predictors of anemia among children. The detailed map of anemia hot spots among children aged 6–59 months and its predictors could assist program planners and decision-makers to design targeted public health interventions.

## Background

Anemia is the commonest public health problem in Low-and Middle-Income Countries (LMICs) [1–3]. Globally, an estimated 1.62 billion people are anemic, of these more than 43% occurred in LMICs particularly in Asia and Africa [4, 5]. Under-five children are highly affected by anemia compared to the general population [6, 7]. According to World Health Organization (WHO), an estimated 293 million under-five children are anemic globally with a prevalence of 47.4% [8]. In Ethiopia, the prevalence of anemia among under-five children was 57% [7, 9]. Anemia during childhood has been linked to developmental delay, recurrent infections, reduced working capacity, poor school performance, and dilated cardiomyopathy [10, 11].

Anemia has a multifactorial etiology and several factors act simultaneously. Nutritional deficiencies such as iron, folate [12, 13], vitamin B12 [14], and vitamin A [15] are the leading causes of anemia among under-five children in LMICs. Besides, diseases like malaria [16], Visceral Leishmaniasis (VL) [17], hookworm [18], schistosomiasis [19], cancer [20], tuberculosis [21], HIV/AIDS [22], and genetic hemoglobinopathies are commonly reported causes of anemia in developing and developed world [23].

Previous studies on anemia among under-five children revealed that residence, maternal educational status, taking drugs for intestinal parasites, child nutrition status (stunting, wasting, and underweight), maternal age, child age, household wealth index, maternal anemia status, child-size at birth, birth order, parity, type of water source, type of toilet facility, type of birth, sex of children, and media exposure were significantly associated with anemia [24–27].

According to studies reported on the prevalence and associated factors of anemia, the prevalence of anemia has significant variation across regions in Ethiopia [7, 28–34]. Therefore, this study aimed to investigate spatial regression analysis of anemia and its associated factors among children aged 6–59 months. The findings of this study will help policymakers, program planners, and other health care programs in guiding health programs and prioritize prevention and intervention programs. Besides, mapping hotspot areas of anemia will provide a deeper understanding of the impacts of already implemented interventions in each region of the country.

## Methods and materials

### Study area, data source, and study period

This study was based on the 2016 Ethiopian Demographic and Health Survey (EDHS) data. The 2016 EDHS was the fourth DHS in Ethiopia, which was conducted every 5 years. The EDHS is mainly aimed to generate updated health and health-related indicators such as maternal mortality, child mortality, family planning, vaccination, and maternal health care service utilization. Ethiopia is administratively divided into nine geographical regions (Tigray, Afar, Amhara, Oromia, Somalia, Benishangul-Gumuz, Southern Nation Nationality, and People’s Region (SNNPR), Gambella, and Harari) and two self-administrative cities (Addis Ababa and Dire Dawa). Each region is subdivided into zones, each zone into Weredas, and each Wereda into Kebeles (which is the lowest administrative unit). A multistage stratified sampling technique was applied to select the study designs. In the first Enumeration Areas (EAs) was randomly selected and in the second on average 28 households per clusters/EAs.

### Sample and population

Hemoglobin testing was carried out among children aged 6–59 months in the selected households using HemoCue rapid testing methodology. For the test, a drop of capillary blood was taken from a child’s fingertip or heel and was drawn into the micro cuvette which was then analyzed using the photometer that displays the hemoglobin concentration. Then, anemic status was determined based on the hemoglobin level. The EDHS has several datasets such as men (MR file), women (IR file), children (KR file), birth (BR file), and household (HR file) datasets. For this study, we used the Kids Record dataset (KR file), and a total weighted sample of 8482 children aged 6–59 months was included.

### Study variables

The dependent variable was the anemia status of children aged 6–59 months, which was categorized into anemic (hemoglobin level < 11 g/dl) and not anemic (hemoglobin ≥11.0 g/dl). It was assessed based on the hemoglobin concentration in blood adjusted for altitude.

In EDHS, before determining a child is anemic or not, hemoglobin adjustment for altitude was done by subtracting or adding the adjusted Hgb value to each individual observed Hgb value.

The Hgb adjustment was made using the formula;

$$Hgb\;adjustment=‐0.0322\;(altitude\;*\;0.0032808)+0.022\;{\left(altitude\;*\;00032808\right)}^{2}$$

The adjustment for altitude was done to take into account the reduction in oxygen saturation of the blood. The independent variables considered in this study were region, residence, maternal age, maternal educational status, household wealth status, media exposure, maternal anemia status, sex of children, type of birth, age of children, size of child at birth, water source, type of toilet facility, parity, birth order, taking drugs for intestinal parasites in the last 6 months, wasting status (Z-scores for Weight-for-Height (WHZ)), underweight status (Z-scores for Weight-for-Age (WAZ)) and stunting status (Z-scores for Height-for-Age (HAZ)).

Stunting is defined as children with height-for-age Z-score (HAZ) <−2SD, wasting is defined as children with weight-for-height Z-score (WHZ) <−2SD, and underweight is defined as children with weight-for-age Z-score (WAZ) <−2SD. Maternal anemia was defined as “mild”, “moderate”, and “severe anemia” when Hgb level ranges 10–10.9 g/dl, 7–9.9 g/dl, and <7 g/dl, respectively.

### Data management and analysis

#### Factors associated with anemia

Data extraction, coding, and analysis were done using Stata version 14 and Arc-GIS version 10.6 statistical software. The weighted data were used for analysis to restore the representativeness of the data. Since the EDHS data has a hierarchical nature, the Intra-class Correlation Coefficient (ICC) was estimated to assess the clustering effect. The ICC indicated that there was a significant clustering effect (ICC>10%). This study was a cross-sectional study and the prevalence of anemia was greater than 10%, and if we reported the odds ratio it could overestimate the association between anemia and the independent variables. In such cases, the prevalence ratio is the best measure of association, and therefore, multilevel Poisson regression analysis with robust variance was fitted to identify predictors of anemia. Variables with a p-value<0.2 in the bi-variable multilevel Poisson regression analysis were considered for the multivariable analysis. Deviance was used to verify model fitness, and a model with the lowest deviance was considered the best-fit model. Finally, the Adjusted Prevalence Ratio (APR) with its 95% confidence interval (CI) was reported, and variables with p value<0.05 in the multivariable analysis were considered as significant predictors of anemia among under-five children.

#### Spatial analysis

The global spatial autocorrelation (Global Moran’s I) was done to assess whether the spatial distribution of anemia among under-five children in Ethiopia was dispersed, clustered, or randomly distributed [35]. Global Moran’s I is a spatial statistic used to measure spatial autocorrelation by taking the entire data set and produce a single output value that ranges from -1 to +1. Moran’s, I value close to −1 indicates that anemia among under-five children is dispersed, whereas Moran’s I close to +1 indicates anemia among under-five children is clustered and if Moran’s I close to 0 revealed that anemia among under-five children is randomly distributed. A statistically significant Moran’s I (p < 0.05) value showed that anemia among under-five children is non-random. The hotspot analysis was done using the Getis-OrdGi* statistics to explore how spatial autocorrelation varies over the study location by calculating GI* statistic for each area. Z-score is computed to determine the statistical significance of clustering, and the p-value is computed for the significance. Statistical output with high GI* indicates "hotspot" whereas low GI* means a "cold spot" [36].

#### Spatial regression analysis

The Ordinary Least Square (OLS) regression and Geographic Weighted Regression (GWR) statistical analysis were employed for exploring the spatial relationship between anemia among under-five children and the explanatory variables. The outcome variable for spatial regression analysis was the percentage of anemia among under-five children at the EA level. A neighborhood or bandwidth is the distance band or the number of neighbors used for each regression equation, it is the most important parameter for spatial regression as it controls the degree of smoothening in the model. The complexity of spatial regression model depends not only by the number of variables in the model but also the bandwidth. There are three choice of band width methods such as AICc, CV and bandwidth parameter. For this study we have used adaptive kernel whose bandwidth was found by minimizing the AICc value.

*Ordinary Least Squares (OLS) regression*. The spatial regression modeling was performed to identify predictors of the spatial heterogeneity of anemia among under-five children. OLS is a global statistical model for testing and explaining the relationship between the dependent and independent variables [37]. It uses a single equation to estimate the relationship between the dependent and independent variables and assumes stationarity or consistent relationship across the study area. The OLS was used as a diagnostic tool and for selecting the appropriate predictors (concerning their relationship with anemia) for the Geographic Weighted Regression (GWR) model [38].

The OLS can automatically check the multicollinearity between independent variables (redundancy among explanatory variables). The multicollinearity was assessed using the Variance Inflation Factor (VIF). If the VIF values are greater than 10 in the OLS model, it indicates the existence of multicollinearity among the explanatory variables and should apply to leave one out an approach based on the VIF values. Besides, the autocorrelation statistic was applied to detect whether there is spatial autocorrelation or clustering of the residuals which violates the assumptions of OLS. The spatial independence of the residuals was assessed with the global spatial autocorrelation coefficient Moran’s I value. The Moran’s I value ranges from +1 (positive autocorrelation) and -1 (negative autocorrelation).

*Geographically Weighted Regression (GWR)*. A local spatial statistical technique that assumes the non-stationarity in relationships/ heterogeneity in the relationship between the dependent and explanatory variables across EAs [38–40]. The GWR analysis is considered when the Koenker statistics is significant (p-value<0.05), which means the relationships between the dependent and the independent variable change from location to location. In the GWR analysis, the coefficients of the explanatory variables take different values across the study area. Mapping the GWR coefficients associated with the explanatory variables, which are produced using the GWR, provides insight for targeted interventions. The corrected Akaike Information Criteria (AICc) and adjusted R-squared for model comparison of OLS (global model) and GWR (local) model. A model with the lowest AICc value and a higher adjusted R-squared value was considered as the best-fitted model for the data.

## Results

### Descriptive results

A total weighted sample of 8482 children aged 6 to 59 months was included. Of these, more than half (51.85%) of the children were males. The majority (43.87%) of the children were in the Oromia region and 7621 (89.85%) were from the rural areas. Nearly half of the mothers (48.76%) were aged 20–29 years, and 5684 (67.02%) of the mothers didn’t attain formal education. About 1988 (23.44%) and 1142 (13.46%) of the mothers fall within the poorest and richest household index quintiles, respectively. Nearly one-third (30.1%) of the children’s mothers were anemic. Regarding children’s nutritional status, about 40.73%, 25.29%, and 9.38% of the children were stunted, underweight and wasted, respectively (Table 1).

**Table 1:** Descriptive characteristics of the study participants in Ethiopia, 2016.

Variables	Weighted frequency	Percentage
**Region**		
Tigray	572	6.75
Afar	83	1.00
Amhara	1657	19.54
Oromia	3722	43.87
Somali	349	4.11
Benishangul-gumuz	90	1.07
SNNPRs	1781	20.99
Gambella	20	0.23
Harari	16	0.19
Addis Ababa	161	1.90
Dire-Dawa	31	0.37
**Residence**		
Rural	7621	89.85
Urban	861	10.15
**Maternal age (years)**		
<20	226	2.67
20–29	4136	48.76
30–39	3335	39.31
≥40	785	9.25
**Maternal education status**		
No	5684	67.02
Primary	2281	26.89
Secondary or higher	516	6.09
**Household wealth status**		
Poorest	1988	23.44
Poorer	1989	23.45
Middle	1823	21.49
Richer	1540	18.15
Richest	1142	13.46
**Media exposure**		
No	5787	68.22
Yes	2695	31.78
**Maternal anemia status**		
Not anemic	5861	69.90
Anemic	2524	30.10
**Sex of children**		
Male	4398	51.85
Female	4084	48.15
**Type of birth**		
Single	8274	97.55
Multiple	208	2.45
**Age of children (months)**		
6–23	2908	34.28
24–59	5574	65.72
**Size of children at birth**		
Small	2177	25.66
Average	3565	42.03
Large	2740	32.31
**Water source**		
Not improved	3841	45.28
Improved	4641	54.71
**Toilet facility**		
Not improved	3297	38.87
Improved	5185	61.13
**Parity**		
1–3	3640	42.91
4–6	2945	34.72
>6	1897	22.37
**Birth order**		
1–3	4118	48.55
4–6	2816	33.20
>6	1548	18.24
**Taking drugs for intestinal parasites in the last 6 months**		
No	7391	87.14
Yes	1091	12.86
**Stunting status**		
Not stunted	5027	59.27
Stunted	3455	40.73
**Underweight**		
No	6337	74.71
Yes	2145	25.29
**Wasting status**		
Not wasted	7687	90.62
Wasted	795	9.38

### Prevalence of anemia among under-five children in Ethiopia

**Fig 1:**
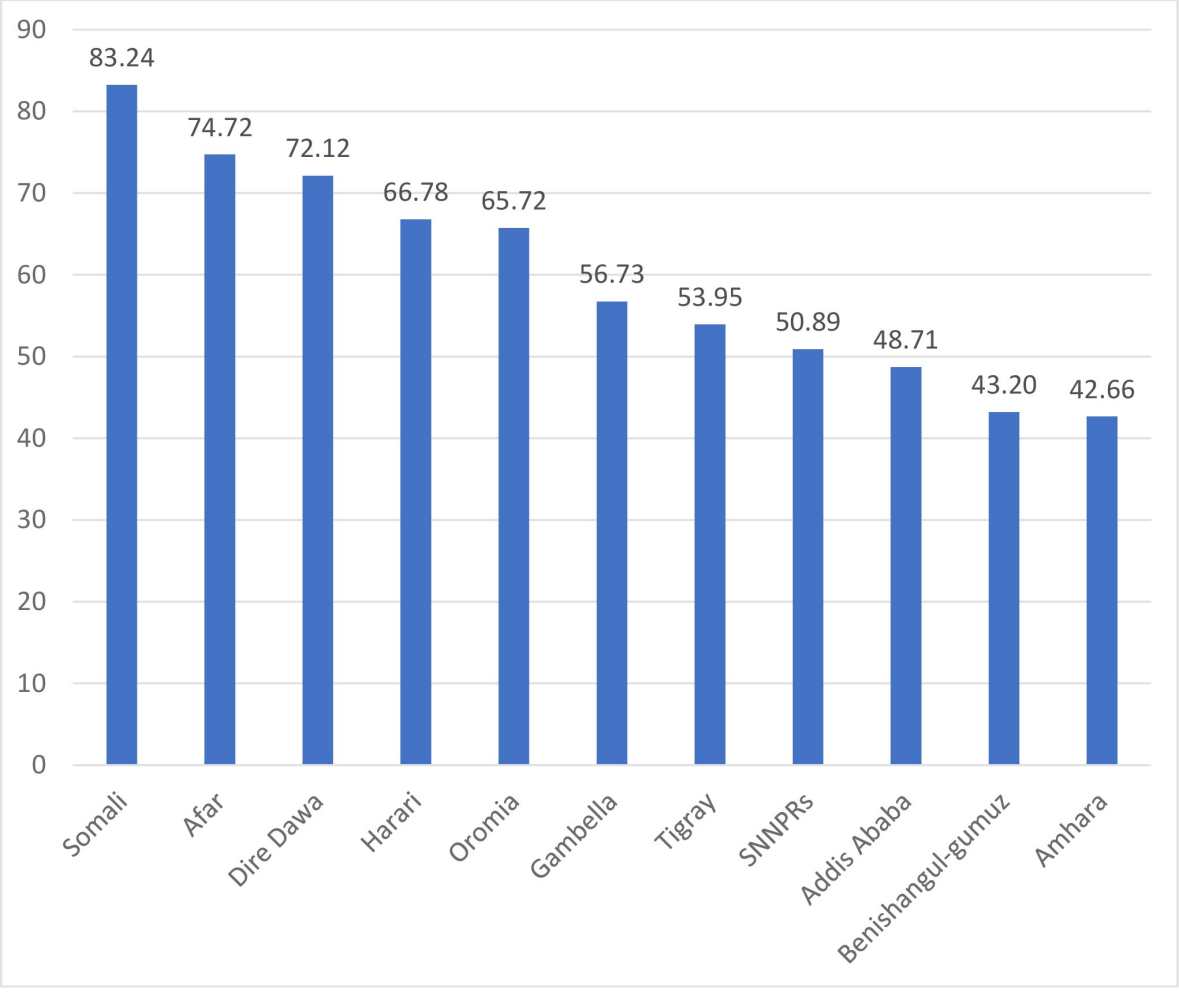
The prevalence of anemia among under-five children across regions in Ethiopia, 2016.

The prevalence of anemia among under-five children was 57.56% (95%CI: 56.50%, 58.61%). The highest prevalence of anemia among under-five children was observed in Somali (83.24%), Afar (74.72%), and Dire Dawa (72.12%) regions. Whereas, the lowest prevalence of anemia was observed in Addis Ababa (48.71%), Benishangul-gumuz (43.20%), and Amhara (42.66%) regions (Fig 1).

### Factors associated with anemia among under-five children

#### Random effect analysis results

In the null model, the ICC value was 18.8% (95% CI: 15.99%, 21.98%), indicated that about 18.8% of the overall variability in anemia was explained by the between cluster variation while the remaining 81.20% was attributed to the individual-level variation. Besides, the Likelihood Ratio (LR) test was (LR test vs. logistic model: X^2^(01) = 512.36, p< 0.0001), which showed that the mixed-effect models were the best-fitted model for this data compared to the standard model.

#### Fixed effect analysis results

In the multivariable mixed-effect Poisson regression with a robust variance; maternal age, maternal education, household wealth index, birth order, maternal anemia, age of children, stunting, and underweight were significantly associated with anemia among under-five children. The prevalence of anemia among children born to mothers aged 30–39 and 40–49 years was decreased by 16% (APR = 0.84, 95% CI: 0.76, 0.92) and 27% (APR = 0.73, 95% CI: 0.65, 0.83) compared to children born to mothers aged less than 20 years, respectively. Children whose mothers did not have formal education had 1.10 times (APR = 1.10, 95% CI: 1.00, 1.20) higher prevalence of anemia than children whose mothers attained secondary education or higher. Children in the poorest household wealth index were 1.17 times (APR = 1.17, 95% CI: 1.06, 1.29) higher prevalence of anemia compared to children in the richest household wealth index. Being the 4^th^-6^th^ and above 6^th^ order of birth increases the prevalence of anemia by 1.08 (APR = 1.08, 95% CI: 1.02, 1.13) and 1.15 (APR = 1.15, 95% CI: 1.07, 1.23) than first to third births. Children born to anemic mothers had 1.24 times (APR = 1.24, 95% CI: 1.19, 1.29) a higher prevalence of anemia compared to children born to non-anemic mothers. The prevalence of anemia among children aged 24–59 months was decreased by 30% (APR = 0.70, 95% CI: 0.68, 0.73) compared to children aged 6–23 months. Stunted children had 1.09 times (APR = 1.09, 95% CI: 1.04, 1.13) a higher prevalence of anemia compared to no-stunted children, and underweight children had 1.07 times (APR = 1.07, 95% CI: 1.03, 1.13) higher prevalence of anemia compared to normal children (Table 2).

**Table 2:** Bi-variable and multivariable mixed-effect robust Poisson regression analysis of anemia among under-five children in Ethiopia, 2016.

Variables	Anemia status		Crude Prevalence Ratio with 95% CI	Adjusted Prevalence Ratio with 95% CI
	No	Yes		
**Residence**				
Urban	608	720	1	1
Rural	2496	3971	1.47 (1.20, 1.79)	0.95 (0.86, 1.04)
**Maternal age**				
<20	61	169	1	1
20–29	1471	2411	0.59 (0.43, 0.81)	0.93 (0.85, 1.01)
30–39	1248	1772	0.53 (0.38, 0.73)	0.84 (0.76, 0.92) *
40–49	324	339	0.40 (0.28, 0.56)	0.73 (0.65, 0.83)**
**Maternal educational status**				
No	1903	3181	1.44 (1.17, 1.76)	1.10 (1.00, 1.20)*
Primary	840	1141	1.29 (1.05, 1.60)	1.08 (0.98, 1.18)
Secondary or above	361	368	1	1
**Household wealth index**				
Poorest	849	2005	2.17 (1.80, 2.60)	1.17 (1.06, 1.29)**
Poorer	568	819	1.50 (1.23, 1.82)	1.03 (0.94, 1.14)
Middle	543	613	1.22 (0.98, 1.49)	0.96 (0.87, 1.07)
Richer	465	518	1.22 (0.99, 1.50)	0.98 (0.89, 1.09)
Richest	679	736	1	1
**Media exposure**				
No	1974	3356	1	1
Yes	1130	1335	0.73 (0.64, 0.82)	0.96 (0.91, 1.01)
**Birth order**				
1–3	1652	2326	1	1
4–6	972	1557	1.09 (0.98, 1.23)	1.08 (1.02, 1.13)**
>6	480	808	1.12 (0.96, 1.29)	1.15 (1.07, 1.23)**
**Maternal anemia status**				
Not anemic	2291	2709	1	1
Anemic	781	1908	1.61 (1.43, 1.81)	1.24 (1.19, 1.29)*
**Child size at birth**				
Small	735	1372	1	1
Average	1372	1944	0.85 (0.75, 0.96)	0.96 (0.92, 1.01)
Large	997	1375	0.80 (0.70, 0.92)	0.97 (0.92, 1.02)
**Water source**				
Not improved	1160	2051	1	1
Improved	1944	2640	0.82 (0.72, 0.93)	0.99 (0.95, 1.04)
**Type of toilet facility**				
Not improved	1195	2369	1	1
Improved	1909	2322	0.69(0.61, 0.78)	0.96 (0.91, 1.01)
**Age of children (months)**				
6–23	3031	4578	1	1
24–59	73	113	0.33 (0.29, 0.37)	0.70 (0.68, 0.73)*
**Taking drugs for intestinal parasites in the last 6 months**				
No	2621	4133	1	1
Yes	483	558	0.81 (0.70, 0.95)	0.98 (0.92, 1.05)
**Stunting status**				
Not stunted	2014	2759	1	1
Stunted	1090	1932	1.35 (1.21, 1.50)	1.09 (1.04, 1.13)**
**Underweight**				
Normal	2416	3283	1	1
Underweight	688	1408	1.51 (1.34, 1.70)	1.07 (1.03, 1.13)*
**Wasting status**				
Normal	2833	4059	1	1
Wasted	271	632	1.17 (1.12, 1.23)	1.03 (0.98, 1.095)

*p-value<0.05

**p-value<0.01

#### Spatial distribution of anemia among under-five children

**Fig 2:**
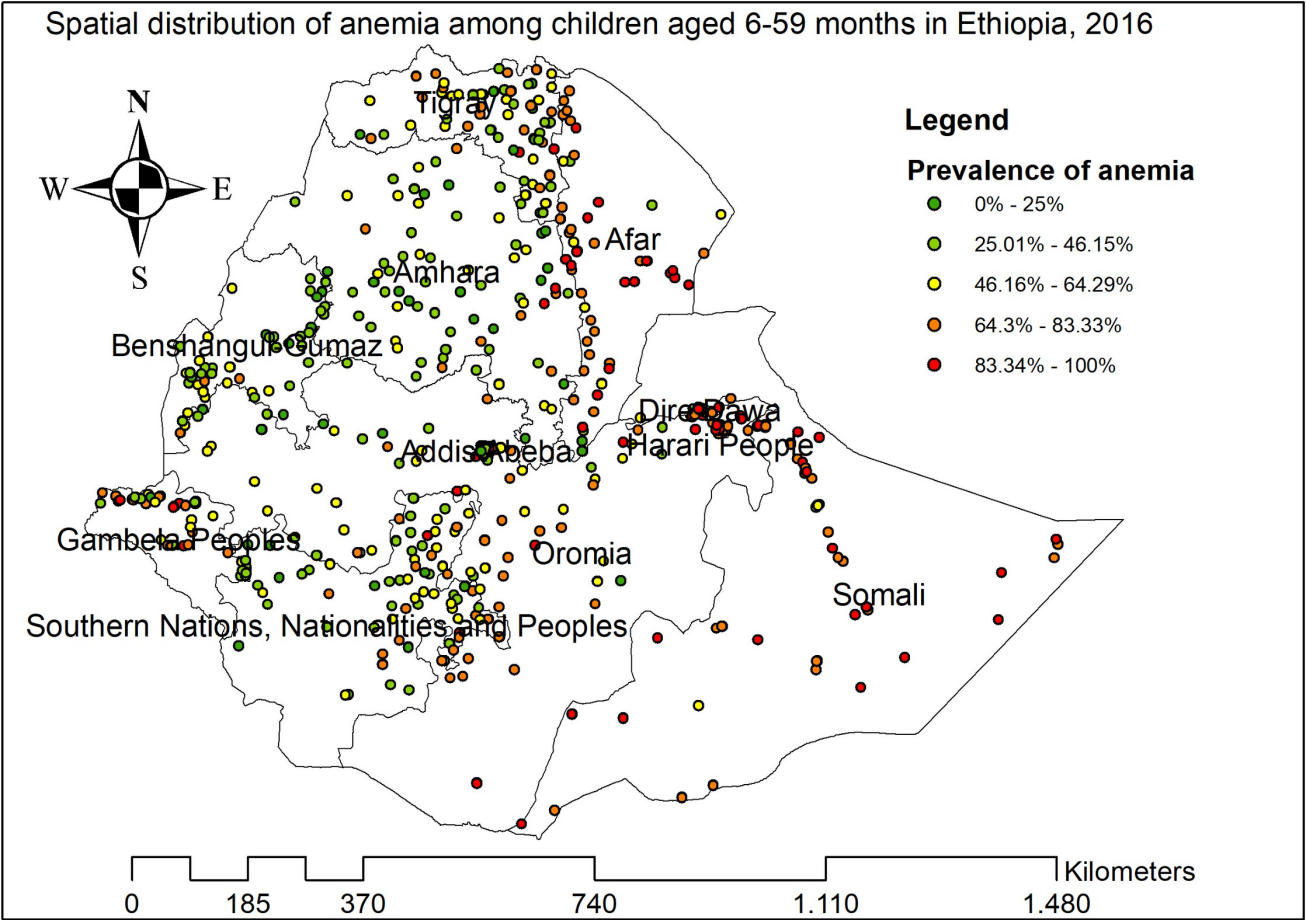
The spatial distribution of anemia among children aged 6–59 months in Ethiopia, 2016.

**Fig 3:**
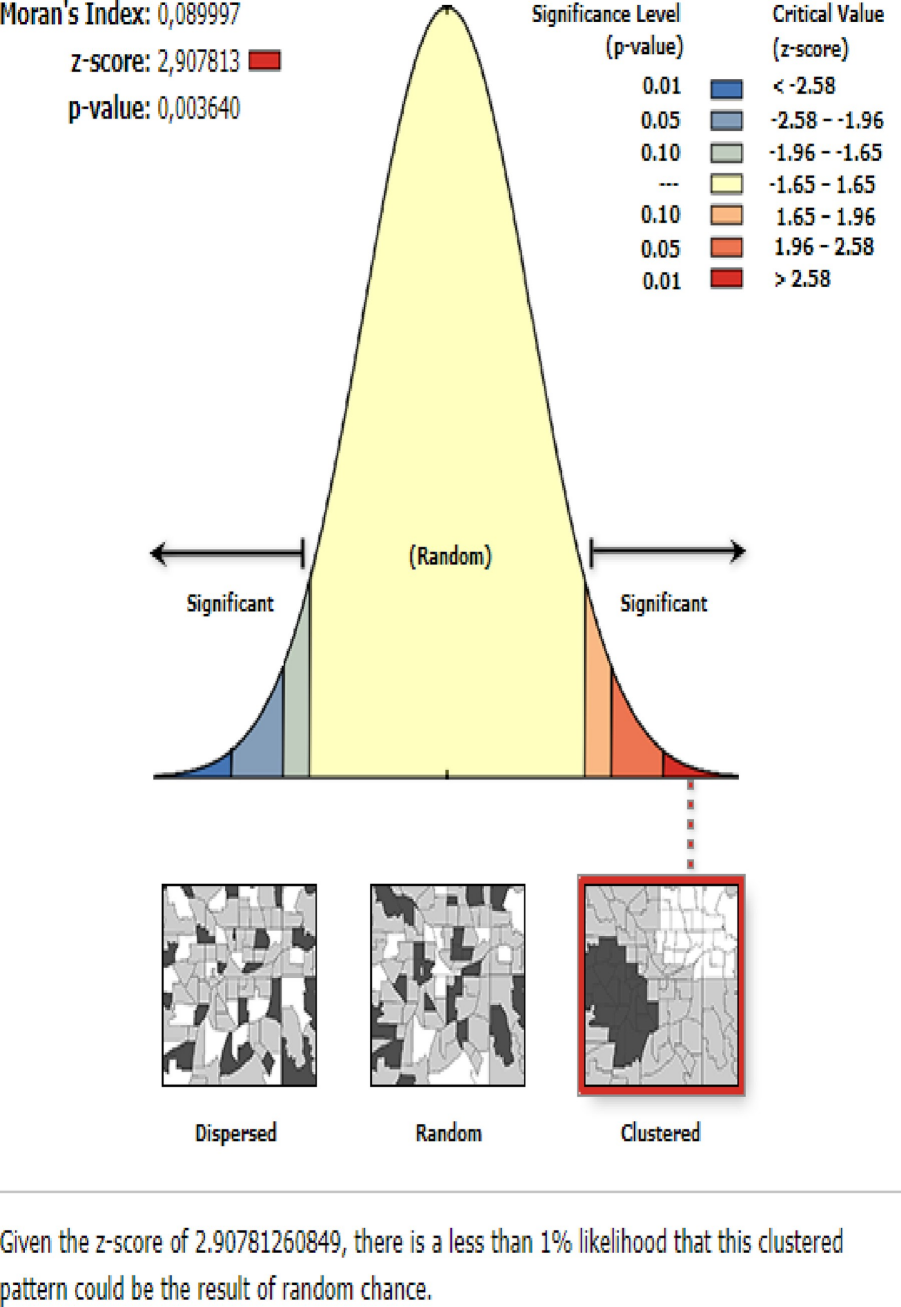
The global spatial autocorrelation analysis of anemia among children aged 6–59 months in Ethiopia, 2016.

**Fig 4:**
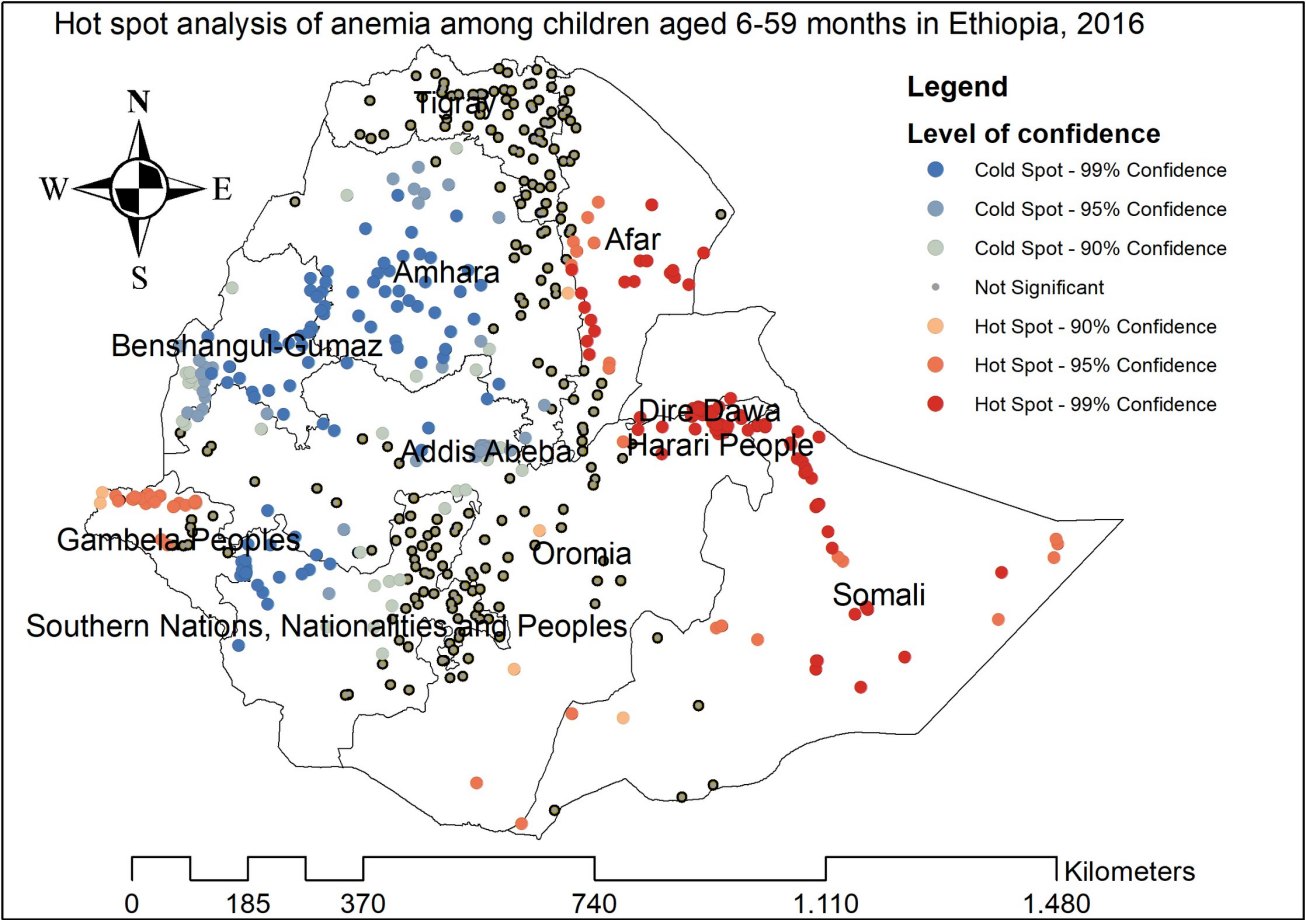
The Getis Ord Gi statistical analysis of hot spots of anemia among children aged 6–59 months in Ethiopia, 2016.

The highest prevalence of anemia among children aged 6–59 months was observed in Afar, Somali, Tigray, Dire Dawa, and east Amhara regions (Fig 2). The spatial distribution of anemia among children aged 6–59 months showed significant spatial variation across the country with a global Moran’s I value of 0.089 (p-value<0.01) (Fig 3). The statistically significant hotspot areas of anemia were identified in the central, west, and east Afar, Somali, Dire Dawa, Harari, and northwest Gambella regions. While significant cold spot areas were detected in the northwest SNNPRs, Addis Ababa, Benishangul-gumuz, central and southwest Amhara regions (Fig 4).

#### The global ordinary least square regression analysis results

The OLS model was calibrated to diagnose multicollinearity among the independent variables and the mean VIF was less than 10. In the OLS analysis, the model explained about 26% (adjusted R^2^ = 0.22) of the variation in anemia among children aged 6–59 months with AICc = -169.06. The Joint F-statistics and Wald statistics were significant (p<0.05), which proves that the model was statistically significant. The spatial distribution of residuals was normally distributed as the Jarque-Bera statistics were non-significant (the residuals were normally distributed) (p = 0.18). The Koenker statistics were statistically significant, indicates that the relationship between the independent variables and the dependent variable was non-stationary or heterogeneous across the study areas. This indicates that GWR should be applied (since the Koenker statistics showed the non-stationarity in the relationship) as it assumes the spatial heterogeneity of the relationship between independent and dependent variables across space. The proportion of women aged 15–19 years, the proportion of women who were anemic, the proportion of women who attained formal education, and the proportion of children aged 6–23 months were significantly associated with the percentage of anemia among children aged 6–59 months in the OLS model (Table 3).

**Table 3:** The Ordinary Least Square (OLS) regression analysis result.

Variable	Coefficient		Robust std-error	Robust t-statistics	Robust probability	VIF
Intercept	0.29		0.049	5.91	0.00001*	----
Proportion of mothers aged 15–19 years	0.27		0.12	2.21	0.027*	1.09
Proportion of mothers who had formal education	-0.01		0.039	-0.26	0.79	2.13
Proportion of women with poverty	0.09		0.03	2.93	0.003*	1.97
Proportion of birth order greater than 6	0.04		0.046	-0.89	0.37	1.60
Proportion of mothers with anemia	0.37		0.04	8.93	0.000001*	1.22
Proportion of children aged 6–23 months	0.30		0.068	4.34	0.00002*	1.06
Proportion of stunted children	0.07		0.048	1.41	0.15	1.22
Proportion of wasted children	0.11		0.098	1.11	0.27	1.15
**Ordinary least square regression Diagnostics**						
Number of observations	615	Adjusted R-squared			0.26	
Joint F-statistics	27.79	Prob(>F), (8,600) degree of freedom			<0.001	
Joint Wald statistics	221.17	Prob (> chi-squared), (8) degree of freedom			< 0.001	
Koenker (BP) statistics	54.94	Prob (> chi-squared), (8) degree of freedom			< 0.001	
Jarque–Bera	3.38	Prob (> chi-squared), (2) degree of freedom			0.18	

VIF: Variance Inflation Factor

#### The geographically weighted regression analysis result

The GWR analysis showed that there was a significant improvement over the global model (OLS). The AICc value decreased from -169.06 (for the OLS model) to -257.41 (for the GWR model). The difference was 88.35 implied that the GWR best explains the spatial heterogeneity of anemia among children aged 6–59 months. Besides, the adjusted R^2^ was 0.38, the model’s ability to explain anemia among children aged 6–59 months has been improved by using GWR since the adjusted R^2^ was 0.38, indicates that GWR improved the model explaining the power of the OLS model by about 12% (Table 4). In the geographically weighted regression analysis, the proportion of women who had formal education, the proportion of women aged 15–19 years, the proportion of women with poverty, the proportion of being birth order above 6^th^, proportion of women who had anemia, proportion of children aged 6–23 months, the proportion of stunted children and proportion of wasted children were considered as explanatory variables in the GWR model since it was significant in the multilevel robust Poisson regression analysis as well as have good R^2^ in the exploratory analysis.

**Table 4:** Model comparison of OLS and GWR model.

Model Comparison parameter	OLS model	GWR Model
AICc	-169.06	-257.41
Adjusted R-squared	0.26	0.38

**Fig 5:**
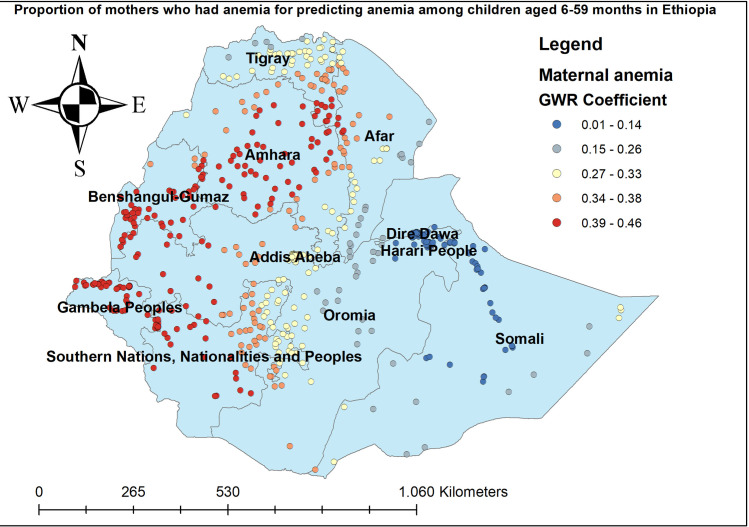
Mothers with anemia GWR coefficients for predicting anemia among children aged 6–59 months in Ethiopia, 2016.

**Fig 6:**
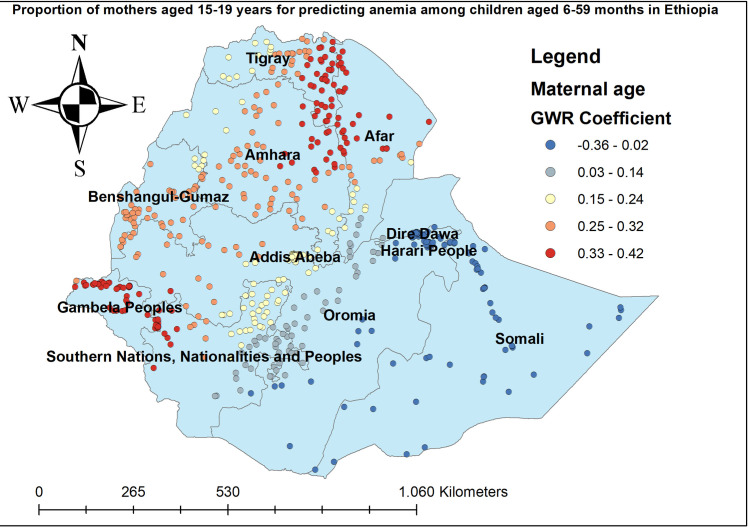
Mothers aged 15–19 years GWR coefficients for predicting anemia among children aged 6–59 months in Ethiopia, 2016.

**Fig 7:**
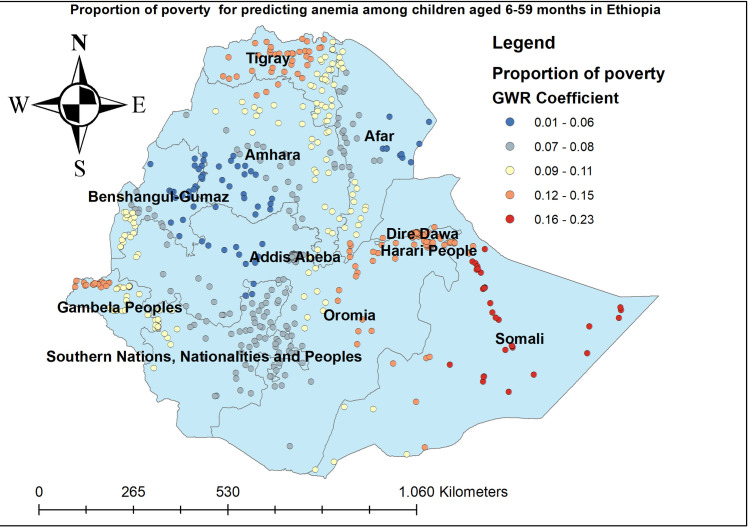
Child coming from a household with poorer or poorest wealth index GWR coefficients for predicting anemia among children aged 6–59 months in Ethiopia, 2016.

**Fig 8:**
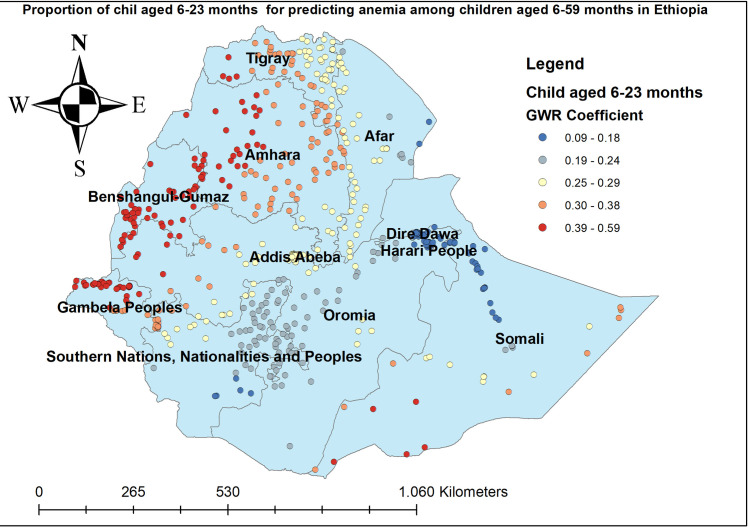
Children aged 6–23 months GWR coefficients for predicting anemia among children aged 6–59 months in Ethiopia, 2016.

The proportion of women who had anemia had a positive relationship with the proportion of anemia among children aged 6–59 months. As the proportion of women who had anemia increased, the percentage of anemia among under-five children increased in the entire Amhara, Benishangul-gumuz, Gambella, and SNNP regions. The geographic area with red-colored points indicates the highest coefficient of the proportion of maternal anemia (Fig 5). The proportion of mothers aged 15–49 years was significantly associated with the increased risk of anemia among children aged 6–59 months, with the highest effect of mothers age observed in southeast Amhara, east Tire, west Afar, Gambella, and southwest SNNP regions (Fig 6). The proportion of mothers in the poorest household wealth status showed strong and positively associated with increased risk of anemia among under-five children in Somali regions (Fig 7). The proportion of children aged 6–23 months had a significant positive association with anemia among children aged 6–59 months (Fig 8).

## Discussion

Anemia among children aged 6–59 months remains a common public health problem in Ethiopia. In this study, the prevalence of anemia among children aged 6–59 months in Ethiopia was 57.56% (95%CI: 56.50%, 58.61%) ranged from 42.66% in the Amhara region to 83.24% in the Somali region. This was higher than a study reported in Ghana [41] and China [42]. Even though the combined strategies particularly iron supplementation and infectious disease management (such as malaria and helminths infections) are being introduced by the WHO to combat anemia, anemia remains a serious health care problem in Ethiopia. Besides, the spatial distribution of anemia among children aged 6–59 months in Ethiopia was non-random and the hotspot areas of anemia were identified in the central, west, and east Afar, Somali, Dire-Dawa, Harari, and northwest Gambella regions. Though numerous interventions have been implemented to reduce anemia among children such as nutritional interventions like food fortification and supplementation [43–45], iron-folate supplementation during pregnancy [46, 47], appropriate breastfeeding practice [48], and improving personal hygiene [49], there was high spatial heterogeneity of anemia across areas.

The potential reason may be due to the long-standing prevalence of severe malnutrition among under-five children, because of insufficient dietary intake of nutrients in Ethiopia [50, 51]. Besides, Ethiopian children’s are highly affected by infectious diseases such as malaria, hookworms, Schistosoma, and visceral leishmaniasis, due to their frequent exposure to poor sanitation and environmental conditions that favor the transmission and spread of parasites [52–54]. In the spatial regression analysis, maternal age, child age, poverty, and maternal anemia was significant predictors of hotspot areas of anemia among under-five children. There is a positive relationship between poverty and hotspots of anemia in Somali regions. Inadequate wealth in the community is associated with an increased risk of intestinal infections such as hookworm, amoebiasis, and ascariasis and these could increase the risk of anemia [55, 56]. Besides, children in the poorest household are prone to undernutrition like lack of folate, iron vitamin B12, and vitamin A [57]. An increased proportion of mothers aged 15–19 years increases the odds of anemia among under-five children in southeast Amhara, east Tigray, west Afar, Gambella, and southwest SNNP regions. The possible explanation might be due to adolescent pregnant mothers are more likely to give low birth weight, preterm or small for gestational age babies [58, 59], in turn, these might contribute to low hemoglobin levels in the blood. The proportion of children aged 6–23 months had a significant positive association with anemia among children aged 6–59 months, this could be due to children aged 6–23 months are at higher risk of exposure to infectious diseases like foreign body aspiration, pneumonia, diarrheal diseases, and intestinal infections because of exposure to complementary feeding, which increases the malabsorption of iron or folate and increases the risk of anemia [60]. In addition, an increased proportion of anemic mothers increases the odds of anemia among children. This could be due to both mothers and children mostly share a common home environment, which involves mutual exposure to a common set of physical, socioeconomic, and dietary conditions [61]. Also, maternal anemia might be associated with poor birth outcomes such as low birth weight and prematurity of the child, which might lead to limited fetal iron stores and the amount of iron secreted by the breast milk might be insufficient for the daily iron requirement of the child [62].

In the multivariable mixed-effect Poisson regression with a robust variance; maternal age, maternal education, household wealth index, birth order, maternal anemia, age of children, stunting, and underweight were significantly associated with anemia among under-five children.

A child born to a mother aged 30–39 years and 40–49 years had decreased prevalence of anemia compared to a child born to a mother aged less than 20 years. These findings were in line with previous studies reported in Ethiopia [63] and Bangladesh [64]. Anemia is a major public health problem worldwide primarily affects women particularly teenage pregnant girls. Adolescents are particularly susceptible because of their rapid growth and associated high iron requirements. So, pregnancy and lactation increase their nutritional demand, in turn, babies born to teenagers are at higher risk of anemia [65]. Children aged 6–23 months are at higher risk of anemia compared to children aged 24–59 months. This is consistent with study findings reported in Ethiopia [66], and Uganda [67]. It could be due to complementary feeding is initiated after 6 months of birth and during this, a child is exposed to contaminated food and malabsorption syndrome, this could increase nutritional deficiency anemia. In addition, an increased iron requirement due to rapid growth, low availability of foods rich in iron, and lack of diet variety. Iron intake is also likely to improve with age as a result of a more varied diet, including the introduction of meat and other iron-containing foods. The prevalence of anemia was higher among children born to anemic mothers compared to children born to non-anemic mothers. It is in line with study findings in Ghana [68], and Bangladesh [64]. This might be due to children born to anemic mothers are more likely to have nutritional deficiencies like folate, iron, vitamin B12, and vitamin A. Besides, anemic mothers might have underlying diseases such as malaria, HIV/AIDS, or other genetic diseases, and these could transmit to the newborn transplacental, which in turn, could result in anemia. Moreover, mothers and children have common nutritional sources and practices.

Another most important significant predictors of anemia among children were maternal education. A child born to a mother who did not have formal education had a higher prevalence of anemia compared to a child born to a mother who attained secondary education or above. It is consistent with study findings reported in Korea [69]. The possible explanation might be due to children born to mothers who did not attain formal education are more likely to consume iron-rich food like meat and poultry compared to children of educated mothers [70]. In addition, educated mothers are more likely to utilize child health services, which can have a positive effect on their children’s health outcomes, and improved mothers’ level of education results in the corresponding improvement in child feeding practice [71]. Educated mothers exclusively breastfeed their children and initiate appropriate complementary feeding after six months of gestation [72].

High birth order was significantly associated with an increased prevalence of anemia among children aged 6–59 months. This is consistent with studies reported in Sub-Saharan Africa [73] and India [62]. This might be due to as the birth order increases there is maternal nutritional depletion and higher-order births are more likely to be low birth weight, this could increase the risk of anemia [74].

In this study, child nutritional status was significantly associated with anemia among children aged 6–59 months. Children who were stunted and/or underweight were more likely to be anemic than their counterparts. This could be due to anemia and malnutrition often share common causes, it is expected that multiple nutrition problems would co-occur in the same individuals. Low intake of iron-rich foods and diminished nutrient absorption caused by changes in the gastrointestinal epithelium in malnourished individuals contribute towards the development of anemia.

## Strength and limitations of the study

This study has several strengths. It is based on the nationally representative DHS data which was weighted and a multilevel model was fitted that enables us to generalize these findings at the national level. Besides, spatial and geographic weighted regression analyses were conducted, the findings can assist the policymakers and program planners to design spatially targeted public health interventions to reduce the incidence of anemia. The findings of this study should be interpreted considering the following limitations. First, important variables such as underlying medical conditions like malaria, HIV/AIDS, visceral leishmaniasis, and hookworm infections, etc were not considered in the analysis as these variables were not collected in EDHS 2016. Also, we are unable to show the cause-effect relationship between the dependent and independent variables as the DHS data has cross-sectional nature. The geographic locations (GPs) of enumeration areas were displaced up to 2 kilometers in urban and 5 kilometers for most enumeration areas in rural and 10 kilometers for 1% of clusters in rural areas for the sake of privacy, could affect the estimated cluster effects in the spatial regression.

## Conclusion

The prevalence of anemia among children aged 6–59 months in Ethiopia was high, and there was a significant spatial variation of anemia among children aged 6–59 months across regions in Ethiopia. Significant hotspot areas of anemia were identified in the central, west, and east Afar, Somali, Dire Dawa, Harari, and northwest Gambella regions. Being a resident in a geographic area with high community poverty, a high proportion of maternal anemia, a high proportion of children aged 6–23 months, and mothers aged 15–19 years increased the risk of experiencing anemia among children aged 6–59 months. Maternal age, maternal education, birth order, maternal anemia, child age, household wealth index, stunting, and underweight were significant predictors of anemia. Therefore, public health interventions targeting hotspot areas of anemia through empowering women with education help reduce the incidence of anemia among children aged 6–59 months.

## Abbreviations

APR: Adjusted Prevalence Ratio
CI: Confidence Interval
CPR: Crude Prevalence Ratio
EDHS: Ethiopian Demographic and Health Survey
ICC: Intra-class Correlation Coefficient
LLR: log-likelihood Ratio
LR: Likelihood Ratio
SSA: Sub-Saharan Africa
WHO: World Health Organization

## References

[1] Lutter C.K., Iron deficiency in young children in low-income countries and new approaches for its prevention. The Journal of nutrition, 2008. 138(12): p. 2523–2528. doi: 10.3945/jn.108.095406 19022983

[2] Balarajan Y., et al., Anaemia in low-income and middle-income countries. The lancet, 2011. 378(9809): p. 2123–2135. doi: 10.1016/S0140-6736(10)62304-5 21813172

[3] Chaparro C.M. and Suchdev P.S., Anemia epidemiology, pathophysiology, and etiology in low-and middle-income countries. Annals of the New York Academy of Sciences, 2019. 1450(1): p. 15. doi: 10.1111/nyas.14092 31008520 PMC6697587

[4] Kassebaum N.J., The global burden of anemia. Hematology/Oncology Clinics, 2016. 30(2): p. 247–308. doi: 10.1016/j.hoc.2015.11.002 27040955

[5] Milman N., Anemia—still a major health problem in many parts of the world! Annals of hematology, 2011. 90(4): p. 369–377. doi: 10.1007/s00277-010-1144-5 21221586

[6] Piel F.B., et al., Global burden of sickle cell anaemia in children under five, 2010–2050: modelling based on demographics, excess mortality, and interventions. PLoS Med, 2013. 10(7): p. e1001484. doi: 10.1371/journal.pmed.1001484 23874164 PMC3712914

[7] Gebreweld A., et al., Prevalence of anemia and its associated factors among children under five years of age attending at Guguftu health center, South Wollo, Northeast Ethiopia. PloS one, 2019. 14(7): p. e0218961. doi: 10.1371/journal.pone.0218961 31276472 PMC6611584

[8] Visser J. and Herselman M., Anaemia in South Africa: the past, the present and the future. South African Journal of Clinical Nutrition, 2013. 26(4): p. 166–167.

[9] Asresie M.B., Fekadu G.A., and Dagnew G.W., Determinants of anemia among children aged 6–59 months in Ethiopia: Further analysis of the 2016 Ethiopian demographic health survey. Advances in Public Health, 2020. 2020.

[10] Stoltzfus R.J., Defining iron-deficiency anemia in public health terms: a time for reflection. The Journal of nutrition, 2001. 131(2): p. 565S–567S. doi: 10.1093/jn/131.2.565S 11160589

[11] Stoltzfus R.J., Iron deficiency: global prevalence and consequences. Food and nutrition bulletin, 2003. 24(4_suppl_1): p. S99–S103. doi: 10.1177/15648265030244S206 17016951

[12] Green R. and Miller J.W. Folate deficiency beyond megaloblastic anemia: hyperhomocysteinemia and other manifestations of dysfunctional folate status. in Seminars in hematology. 1999. [Sheboygan, Wis.]: Grune & Stratton,[c1964–. 9930568

[13] Irwin J. and Kirchner J.T., Anemia in children. American family physician, 2001. 64(8): p. 1379. 11681780

[14] Stabler S.P., Vitamin B12 deficiency. New England Journal of Medicine, 2013. 368(2): p. 149–160. 10.1056/NEJMcp1113996 23301732

[15] Semba R.D. and Bloem M., The anemia of vitamin A deficiency: epidemiology and pathogenesis. European journal of clinical nutrition, 2002. 56(4): p. 271–281. doi: 10.1038/sj.ejcn.1601320 11965502

[16] Ekvall H., Malaria and anemia. Current opinion in hematology, 2003. 10(2): p. 108–114. doi: 10.1097/00062752-200303000-00002 12579035

[17] Goto Y., et al., Prevalence, severity, and pathogeneses of anemia in visceral leishmaniasis. Parasitology research, 2017. 116(2): p. 457–464. doi: 10.1007/s00436-016-5313-x 27822583

[18] Roche M. and Layrisse M., The nature and causes of “hookworm anemia”. The American journal of tropical medicine and hygiene, 1966. 15(6_Part_2): p. 1032–1102. 5334793

[19] Friedman J.F., Kanzaria H.K., and McGarvey S.T., Human schistosomiasis and anemia: the relationship and potential mechanisms. Trends in parasitology, 2005. 21(8): p. 386–392. doi: 10.1016/j.pt.2005.06.006 15967725

[20] Spivak J.L. Cancer-related anemia: its causes and characteristics. in Seminars in oncology. 1994. 8202724

[21] de Mendonça E.B., et al., Anemia in tuberculosis cases: A biomarker of severity? Plos one, 2021. 16(2): p. e0245458. doi: 10.1371/journal.pone.0245458 33529195 PMC7853529

[22] Volberding P.A., et al., Anemia in HIV infection: clinical impact and evidence-based management strategies. Clinical infectious diseases, 2004. 38(10): p. 1454–1463. doi: 10.1086/383031 15156485

[23] Steinberg M.H. and Adams J., Thalassemic hemoglobinopathies. The American journal of pathology, 1983. 113(3): p. 396. 6359893 PMC1916350

[24] Pasricha S.-R., et al., Determinants of anemia among young children in rural India. Pediatrics, 2010. 126(1): p. e140–e149. doi: 10.1542/peds.2009-3108 20547647

[25] Gebreegziabiher G., Etana B., and Niggusie D., Determinants of anemia among children aged 6–59 months living in Kilte Awulaelo Woreda, northern Ethiopia. Anemia, 2014. 2014. doi: 10.1155/2014/245870 25302116 PMC4180192

[26] Khan J.R., Awan N., and Misu F., Determinants of anemia among 6–59 months aged children in Bangladesh: evidence from nationally representative data. BMC pediatrics, 2016. 16(1): p. 1–12. doi: 10.1186/s12887-015-0536-z 26754288 PMC4707771

[27] Kawo K.N., Asfaw Z.G., and Yohannes N., Multilevel analysis of determinants of anemia prevalence among children aged 6–59 months in Ethiopia: classical and Bayesian approaches. Anemia, 2018. 2018. doi: 10.1155/2018/3087354 29973986 PMC6008921

[28] Anteneh Z.A. and Van Geertruyden J.-P., Spatial variations and determinants of anemia among under-five children in Ethiopia, EDHS 2005–2016. Plos one, 2021. 16(4): p. e0249412. doi: 10.1371/journal.pone.0249412 33793640 PMC8016260

[29] Ejigu B.A., Wencheko E., and Berhane K., Spatial pattern and determinants of anaemia in Ethiopia. PloS one, 2018. 13(5): p. e0197171. doi: 10.1371/journal.pone.0197171 29775472 PMC5959194

[30] Hailegebreal S., et al., Spatio-temporal distribution and associated factors of anaemia among children aged 6–59 months in Ethiopia: a spatial and multilevel analysis based on the EDHS 2005–2016. BMJ open, 2021. 11(8): p. e045544. doi: 10.1136/bmjopen-2020-045544 34404697 PMC8372819

[31] Tezera R., et al., Prevalence of anemia among school-age children in Ethiopia: a systematic review and meta-analysis. Systematic reviews, 2018. 7(1): p. 1–7. doi: 10.1186/s13643-017-0670-9 29793528 PMC5968474

[32] Addis Alene K. and Mohamed Dohe A., Prevalence of anemia and associated factors among pregnant women in an urban area of Eastern Ethiopia. Anemia, 2014. 2014. 10.1155/2014/561567 PMC4158560 25215230

[33] Assefa S., Mossie A., and Hamza L., Prevalence and severity of anemia among school children in Jimma Town, Southwest Ethiopia. BMC hematology, 2014. 14(1): p. 1–9. doi: 10.1186/2052-1839-14-3 24433408 PMC3896819

[34] Gebrie A. and Alebel A., A systematic review and meta-analysis of the prevalence and predictors of anemia among children in Ethiopia. African Health Sciences, 2020. 20(4): p.: 2007–21. doi: 10.4314/ahs.v20i4.59 34394267 PMC8351872

[35] Chen Y., New approaches for calculating Moran’s index of spatial autocorrelation. PloS one, 2013. 8(7): p. e68336. doi: 10.1371/journal.pone.0068336 23874592 PMC3709922

[36] Tsai P.-J., et al., Spatial autocorrelation analysis of health care hotspots in Taiwan in 2006. BMC Public Health, 2009. 9(1): p. 464. doi: 10.1186/1471-2458-9-464 20003460 PMC2799414

[37] Shrestha, P.M., Comparison of ordinary least square regression, spatial autoregression, and geographically weighted regression for modeling forest structural attributes using a Geographical Information System (GIS). Remote Sensing (RS) Approach. Thesis. Canada: University of Calgary. http://people.ucalgary.ca/~mcdermid/Docs/Theses/Shrestha_2006.pdf (Accessed October 30, 2012), 2006.

[38] Nazeer M. and Bilal M., Evaluation of ordinary least square (OLS) and geographically weighted regression (GWR) for water quality monitoring: A case study for the estimation of salinity. Journal of Ocean University of China, 2018. 17(2): p. 305–310.

[39] Foody G., Geographical weighting as a further refinement to regression modelling: An example focused on the NDVI–rainfall relationship. Remote sensing of Environment, 2003. 88(3): p. 283–293.

[40] Fotheringham A.S., Brunsdon C., and Charlton M., Geographically weighted regression: the analysis of spatially varying relationships. 2003: John Wiley & Sons.

[41] Shenton L.M., Jones A.D., and Wilson M.L., Factors Associated with Anemia Status Among Children Aged 6–59 months in Ghana, 2003–2014. Matern Child Health J, 2020. 24(4): p. 483–502. doi: 10.1007/s10995-019-02865-7 32030533 PMC7078144

[42] Li H., et al., Anemia prevalence, severity and associated factors among children aged 6–71 months in rural Hunan Province, China: a community-based cross-sectional study. BMC Public Health, 2020. 20(1): p. 989. doi: 10.1186/s12889-020-09129-y 32576157 PMC7310416

[43] Getahun Z., et al., Review of the status of malnutrition and trends in Ethiopia. The Ethiopian Journal of Health Development, 2001. 15(2).

[44] Laillou A., et al., Vitamin A supplementation and estimated number of averted child deaths in Ethiopia: 15 years in practice (2005–2019). Maternal & child nutrition, 2021. 17(3): p. e13132. 33336556 10.1111/mcn.13132 PMC8189216

[45] Abeshu M. and Geleta B., The role of fortification and supplementation in mitigating the ‘hidden hunger’. J Nutr Sci, 2016. 6(459): p. 2.

[46] Molla T., et al., Factors associated with adherence to iron folate supplementation among pregnant women in West Dembia district, northwest Ethiopia: a cross sectional study. BMC Research Notes, 2019. 12(1): p. 1–6. doi: 10.1186/s13104-018-4038-6 30612583 PMC6322306

[47] Sendeku F.W., Azeze G.G., and Fenta S.L., Adherence to iron-folic acid supplementation among pregnant women in Ethiopia: a systematic review and meta-analysis. BMC pregnancy and childbirth, 2020. 20(1): p. 1–9. doi: 10.1186/s12884-020-2835-0 32131751 PMC7057669

[48] Alebel A., et al., Exclusive breastfeeding practice in Ethiopia and its association with antenatal care and institutional delivery: a systematic review and meta-analysis. International breastfeeding journal, 2018. 13(1): p. 1–12. doi: 10.1186/s13006-018-0173-x 30026786 PMC6048887

[49] Tamiru D., et al., Enhancing personal hygiene behavior and competency of elementary school adolescents through peer-led approach and school-friendly: A quasi-experimental study. Ethiopian journal of health sciences, 2017. 27(3): p. 245–254. doi: 10.4314/ejhs.v27i3.6 29217923 PMC5614995

[50] Bhutta Z.A., et al., Severe childhood malnutrition. Nature reviews Disease primers, 2017. 3(1): p. 1–18. doi: 10.1038/nrdp.2017.67 28933421 PMC7004825

[51] Fabunmi T., et al., Nutrient intakes and nutritional status of mothers and their under-five children in a rural community of Oyo state, Nigeria. International Journal of Child Health and Nutrition, 2013. 2(1): p. 39–49.

[52] Smits H.L., Prospects for the control of neglected tropical diseases by mass drug administration. Expert Review of Anti-infective Therapy, 2009. 7(1): p. 37–56. doi: 10.1586/14787210.7.1.37 19622056

[53] Wilson M.E., Geography of infectious diseases. Infectious Diseases, 2010: p. 1055.

[54] Greenwood B., The epidemiology of malaria. Annals of Tropical Medicine & Parasitology, 1997. 91(7): p. 763–769. 9625932 10.1080/00034989760518

[55] O’lorcain P. and Holland C., The public health importance of Ascaris lumbricoides. Parasitology, 2000. 121(S1): p. S51–S71. doi: 10.1017/s0031182000006442 11386692

[56] Nyarango R.M., et al., The risk of pathogenic intestinal parasite infections in Kisii Municipality, Kenya. BMC public health, 2008. 8(1): p. 1–6. doi: 10.1186/1471-2458-8-237 18620608 PMC2478685

[57] Rohner F., et al., Prevalence and public health relevance of micronutrient deficiencies and undernutrition in pre-school children and women of reproductive age in Cote d’Ivoire, West Africa. Public Health Nutrition, 2014. 17(9): p. 2016–2028. doi: 10.1017/S136898001300222X 24171836 PMC11107373

[58] Ren A., et al., Low first-trimester hemoglobin and low birth weight, preterm birth and small for gestational age newborns. International Journal of Gynecology & Obstetrics, 2007. 98(2): p. 124–128. doi: 10.1016/j.ijgo.2007.05.011 17585914

[59] Chang S.-C., et al., Characteristics and risk factors for adverse birth outcomes in pregnant black adolescents. The Journal of Pediatrics, 2003. 143(2): p. 250–257. doi: 10.1067/S0022-3476(03)00363-9 12970642

[60] Wang F., et al., Age of complementary foods introduction and risk of anemia in children aged 4–6 years: a prospective birth cohort in China. Scientific reports, 2017. 7(1): p. 1–8. doi: 10.1038/s41598-016-0028-x 28333130 PMC5363060

[61] Woldie H., Kebede Y., and Tariku A., Factors associated with anemia among children aged 6–23 months attending growth monitoring at Tsitsika Health Center, Wag-Himra Zone, Northeast Ethiopia. Journal of nutrition and metabolism, 2015. 2015. 10.1155/2015/928632 PMC4461778 26106486

[62] Sinha N., Deshmukh P.R., and Garg B.S., Epidemiological correlates of nutritional anemia among children (6–35 months) in rural Wardha, Central India. Indian J Med Sci, 2008. 62(2): p. 45–54. doi: 10.4103/0019-5359.39366 18319531

[63] Habte D., et al., Maternal risk factors for childhood anaemia in Ethiopia. African journal of reproductive health, 2013. 17(3): p. 110–118. 24069773

[64] Rahman M.S., et al., Association between malnutrition and anemia in under-five children and women of reproductive age: Evidence from Bangladesh Demographic and Health Survey 2011. PLoS One, 2019. 14(7): p. e0219170. doi: 10.1371/journal.pone.0219170 31269082 PMC6609031

[65] Molina Cartes R. and González Araya E., Teenage pregnancy. Endocr Dev, 2012. 22: p. 302–31. doi: 10.1159/000326706 22846537

[66] Takele K., Zewotir T., and Ndanguza D., Risk factors of morbidity among children under age five in Ethiopia. BMC Public Health, 2019. 19(1): p. 942. doi: 10.1186/s12889-019-7273-4 31307433 PMC6631490

[67] Menon M.P. and Yoon S.S., Prevalence and Factors Associated with Anemia Among Children Under 5 Years of Age—Uganda, 2009. Am J Trop Med Hyg, 2015. 93(3): p. 521–6. doi: 10.4269/ajtmh.15-0102 26055748 PMC4559690

[68] Parbey P.A., Tarkang E., and Manu E., Risk Factors of Anaemia among Children under Five Years in the Hohoe Municipality, Ghana: A Case Control Study. 2019. 2019: p. 2139717. doi: 10.1155/2019/2139717 31341677 PMC6614984

[69] Choi H.-J., et al., Effects of maternal education on diet, anemia, and iron deficiency in Korean school-aged children. BMC public health, 2011. 11(1): p. 1–8. 22087564 10.1186/1471-2458-11-870 PMC3250969

[70] Degefa, N., et al., Sick Child Feeding Practice and Associated Factors among Mothers of Children Less Than 24 Months Old, in Burayu Town, Ethiopia. 2019. 2019: p. 3293516. 10.1155/2019/3293516 PMC6942812 31929806

[71] Asif A.M. and Akbar M., Inequalities in child health care in Pakistan: measurement and decomposition analysis of maternal educational impact. Public Health, 2020. 183: p. 94–101. doi: 10.1016/j.puhe.2020.03.029 32470697

[72] Mathur N.B. and Dhingra D., Breastfeeding. Indian J Pediatr, 2014. 81(2): p. 143–9. doi: 10.1007/s12098-013-1153-1 23904066

[73] Obasohan P.E., et al., A Scoping Review of the Risk Factors Associated with Anaemia among Children Under Five Years in Sub-Saharan African Countries. Int J Environ Res Public Health, 2020. 17(23). doi: 10.3390/ijerph17238829 33261060 PMC7731158

[74] Buitrón D., Hurtig A.K., and San Sebastián M., [Nutritional status of Naporuna children under five in the Amazon region of Ecuador]. Rev Panam Salud Publica, 2004. 15(3): p. 151–9. doi: 10.1590/s1020-49892004000300003 15096286

